# Mediated representations of violence against women in the mainstream news in Australia

**DOI:** 10.1186/s12889-019-6793-2

**Published:** 2019-05-03

**Authors:** Georgina Sutherland, Patricia Easteal, Kate Holland, Cathy Vaughan

**Affiliations:** 1 Centre for Mental Health, Melbourne School of Population and Global Health, University of Melbourne, Parkville, Australia; 2 Faculty of Business, Government and Law, University of Canberra, ACT, Bruce, Australia; 3 Senior Research Fellow, News and Media Research Centre, Faculty of Arts and Design, University of Canberra, ACT, Bruce, Australia; 4 Gender and Women’s Health, Centre for Health Equity, Melbourne School of Population and Global Health, University of Melbourne, Parkville, VIC Australia

**Keywords:** Violence against women, News, Media, Australia

## Abstract

**Background:**

How the mainstream news media report violence against women is significant if levels of violence are to be reduced and ultimately eliminated. Media reporting is an important indicator through which to measure progress towards shifting social and cultural norms that reinforce or challenge the place of violence against women in our society. The current study, therefore, aimed to establish a baseline picture of the extent and nature of reporting of violence against women by the mainstream Australian news media.

**Methods:**

Descriptive and content analysis of media reports on violence against women that were collected over four months in three states of Australia. Reports were from newspapers, broadcast (television and radio) and online news sites.

**Results:**

Coverage of violence against women in the mainstream news media was extensive. Explicitly situating violent experiences for women within a broader social context was infrequent. Few news reports included information for women on where to seek help. Additionally, news reports rarely elevated the voices of survivors, advocates and other experts, with a disproportionate emphasis on law enforcement, political and criminal justice perspectives.

**Conclusions:**

Despite readiness among journalists and readers to engage in news about violence against women, reporting that promotes public understanding of the issue is not always the norm.

## Background

Violence against women is now recognised as one of the most serious challenges to health and social inclusion for women and girls worldwide [1]. Defined as ‘any act of violence that results in, or is likely to result in, physical, sexual or psychological harm or suffering to women’ [2], it is a prevalent and pervasive issue with far-reaching individual and community impacts. In Australia, recent estimates indicate that one in three women has experienced physical violence since the age of 15, and one in five has experienced sexual violence [3]. Most women experience violence perpetrated by someone they know, most often a current or former male partner [4]. Harms from violence are wide-ranging and include deleterious effects on physical and mental health. Children exposed to violence can experience a range of social, behavioural, emotional and cognitive problems that often persist into adulthood [5]. The estimated cost to the Australian economy of violence against women is more than $20 billion annually [6].

In recognition of these significant social and economic costs, Australia was one of the first countries to develop a comprehensive national strategic approach to primary prevention [7]. ‘*Change the Story’* report reinforces the direction outlined in the Commonwealth Government’s *National Plan to Reduce Violence Against Women and their Children 2010–2022* [8] by recognising that the underlying causes of violence against women are rooted in ongoing social and cultural norms characterised by unequal value afforded to men and women. It follows, therefore that prevention actions must target the dynamic social conditions in which we live [7]. Within these policy documents, it is unsurprising that media is identified as a key priority area.

News media, especially, is thought to play a critical role in shaping public opinion [9]. The way the news is framed including how individuals and events are portrayed can influence personal, political, policy and social justice responses [10–13]. While there is increasing emphasis on the potential for media to play a role in the prevention of violence against women [14], past research suggests the content of news reports frequently misrepresents the issue. For instance, while there are several ways to embed and promote understanding of the social constructions of male perpetrated violence within news coverage (e.g., by acknowledging underlying societal and gender-based factors), research has found that few media reports do so. A body of research work shows that instead, there is a tendency for media reports of violence against women to use event-based reporting or ‘episodic’ framing [15–21]. News coverage that focuses on discrete incidents or events located at specific places and times (episodic framing) tends to elicit individualistic rather than societal attributions of responsibility [22, 23], thus obscuring from its audience the notion that violence against women is a systemic social problem.

Despite growing interest in analysing media portrayals of violence against women, much of the research to date has exclusively focused on news coverage of high-profile stories [24–27] or retrospectively selected new stories about one type of violence only, most often homicide [15, 16, 28–30]. Prior research is also dominated by media analyses of newspaper reporting [31]. While this is likely because newspapers offer researchers an accessible way to monitor media via text-based electronic, archival collections, such as Factiva, their relevance as a key newsmaker is uncertain in the age of the 24-h news cycle where the volume and types of media available have expanded exponentially.

Our study, therefore, makes an important contribution to this burgeoning literature in several ways. First, we were deliberately broad in scope by prospectively monitoring all news reports on violence against women. Second, we analysed reports across multiple media platforms including large circulation city-based newspapers, smaller local newspapers, broadcast news - both television and radio - and a selection of online news sites. Third, news reports were drawn from media in three states of Australia over a four-month time frame. Media reporting is an important indicator of community attitudes and beliefs about violence against women and thus a critical site through which to measure progress towards shifting social norms that reinforce or exacerbate it [7]. The current study, therefore, aimed to establish a baseline picture of the extent and nature of reporting of violence against women by the mainstream Australian news media.

## Methods

We examined media reports on violence against women from print, radio and television news in three states of Australia (New South Wales, NSW; South Australia, SA and Queensland, QLD) over a four-month period (February 22 to June 22, 2015). These three states provided a degree of geographic and demographic diversity that broadly represents media coverage in Australia. Although all three states are populous and highly urbanised, NSW has large regional cities with major media outlets, while QLD is more de-centralised with much larger rural and remote regions without dedicated media channels. SA was included because unlike other capital cities in Australia, Adelaide (the capital of SA) only has one major metropolitan daily newspaper. Four months of monitoring was considered sufficient to account for potential seasonal effects on reporting. News reports were collected from online news sites for a shorter period, beginning on March 22 and ending May 22, 2015. The first few days of monitoring online news (February 22–24) indicated a high volume of reports including inadvertent capture of reports outside the scope of the study (e.g., news reports on Islamic State and Syria/Levant). As a result, we refined our search terms and reduced the time period for online news data collection. Online news reports collected in the first few days of monitoring were excluded from the final analytic sample. Below, we briefly describe the methods used to identify, retrieve and select news reports.

We used a media monitoring and retrieval service (iSentia) to locate eligible news reports. iSentia tracks and monitors news published in newspapers, magazines, television, radio, internet and social networking sites. While primarily engaged by private companies to monitor brand exposure in media, there are also experienced in supporting research of this kind [32].

In total, iSentia searched 77 newspapers across the three states including all major metropolitan daily newspapers, one national newspaper, a selection of suburban newspapers in SA and QLD and a selection of daily and non-daily regional newspapers in NSW. Regional and suburban newspapers were selected to reflect a diverse range of geographic locations. All television and radio networks were monitored for broadcast news reports. This included 96 AM and FM radio stations and more than 50 free-to-air or pay-for-view television stations. Seven online news sites were selected for inclusion in the study, representing some of the most popular online news sites in Australia at the time (derived by total visits, 2012–14, data courtesy of Experian Marketing Services Australia).

iSentia selected eligible reports from a list of key search terms and string phrases relevant to the topic of violence against women. This included, for example, ‘violence’, ‘assault,’ ‘rape,’ ‘domestic,’ ‘homicide/murder’, combined with ‘women, female, wife or partner.’ Newspaper articles were provided as complete digital press clippings. Television and radio reports were provided as ‘broadcast summaries’ that included the source and a précis of its content. Online news reports were provided as a link and downloaded for analysis.

Across all the media outlets only the first report was included (thereby excluding syndicated or duplicate reports) unless any subsequent reports contained new information to that which was originally printed or broadcast. For each unique news report, *iSentia* extracted a range of descriptive information relevant to each media report that included, for example, the date of publication or broadcast, placement, duration and type of media outlet.

### Data analysis and coding

Data analysis was undertaken in two stages. First, we used simple frequencies and percentages to summarise all media reports captured in the four-month study period including an overview by media type: newspaper, broadcast and online news. Second, 10% of all reports, stratified by media type (i.e. print, broadcast and online), were randomly selected for in-depth content analysis. We used descriptive statistics and chi-square tests to explore information about the nature of news media reports. We performed all analyses using Stata version 13.

For the content analysis, each report was coded using a purpose designed coding frame that included: the primary news frame (episodic or thematic), the types of violence most frequently reported, whether news perpetuated common myths and misunderstandings, directly or indirectly blamed victims of violence or excused the behaviour of perpetrators and sources used to inform the story.

Three research staff coded the elements described above. A detailed coding instruction manual was developed and used by the coders to ensure consistency of coding decisions. In addition, we held two training sessions: the first prior to the commencement of coding in which each question in the coding frame was discussed using examples from news reports collected for the study. The second was held after approximately 20% of the coding was complete to check for consistency. Coding difficulties and uncertainties that arose thereafter were discussed by the research team and resolved by consensus.

## Results

### Extent of media coverage

A total of 4516 news reports on violence against women were identified: 1870 (41.4%) were radio broadcasts, 1332 (29.5%) were online news reports, 929 (20.6%) were newspaper articles and 385 (8.5%) were from television. Taking syndication into account, these reports appeared in, or were broadcast more than 15,000 times during the four-month study period.

### Media characteristics

Table 1 provides an overview of media characteristics by media type. Newspaper reports typically appeared in the major metropolitan daily papers and were in the general news sections. A minority appeared on the front page. Article length ranged from a 27-word report in a suburban weekly paper to a three-page feature piece in the Sunday Life section of the *Sun-Herald* in Sydney. Most reports broadcast on radio were within dedicated news bulletins broadcast on morning ‘talk back’ AM radio stations and were of a short duration (< 30 s). News reports on television were also typically short segments (30 s to two minutes) broadcast during the evening news bulletin. Only a minority appeared on current affairs-style television programs. There was a large volume of news reports online, averaging 166 per week. *abc.com.au* – the online platform of Australia’s government owned national news service hosted the most news reports on violence against women, closely followed by *news.com.au**.*

**Table 1:** Media characteristics by media type

NEWSPAPERS (*n* = 929)	
Average	58 newspaper articles per week
Location	70% NSW, 11% QLD, 8% SA, 11% National; 43% metropolitan
Placement	7% front page, 93% body of paper
Type	82% general news, 3% letters, 3% feature articles, 2% editorials
Size	Range = 27 to 2715 words
RADIO (*n* = 1332)	
Average	177 radio broadcasts per week
Location	60% NSW, 24% QLD, 15% SA, 1% National; 77% metropolitan
Station	83% on AM radio
Type	74% news bulletins, 26% other programming
Duration	Range = < 30 s to 20 min
TELEVISION (*n* = 385)	
Average	24 television broadcasts per week
Location	49% NSW, 27% QLD, 13% SA, 11% National; 63% metropolitan
Station	89% commercial/private, 11% government owned
Type	95% news bulletins, 5% current affairs style programming
Duration	Range = < 15 s to 9 min
ONLINE (*n* = 1332)	
Average	166 online news items per week
Hosted by	76% commercial/private, 24% government owned
Type	45% news site, 55% print affiliated site
Audience	Range = 16,259 to 913,860 unique visitors

Results indicated both weekly and monthly variation in the frequency of reporting. Reporting on violence against women declined over the weekend (Saturday and Sunday) in comparison to weekdays (Monday to Friday). Although this pattern was observed across all media platforms it was most prominent for radio broadcasts. There was also monthly variation with a particularly high volume captured in the second month of the study period (23 March to 22 April). In this month, there were two high profile criminal cases: the murders of school student Masa Vukotic in the Melbourne suburb of Doncaster and school teacher Stephanie Scott in the NSW regional town of Leeton. While there was rarely more than one news report per incident, media reports in relation to these two cases alone accounted for one quarter of all media coverage in the four-month study period (26%, *n* = 1112).

### Nature of media reporting

Of the 464 news reports selected for coding, 20 were excluded because they were primarily about child abuse not in the context of family violence. Our final analytic sample, therefore included 93 newspaper articles, 221 broadcast reports (including 188 radio and 33 television reports) and 130 online news reports.

#### Social context

News reports were coded for whether the primary frame was episodic, thematic or a combination approach. Episodic news reports were defined as those that focused on telling the story of an incident of violence; either past or present. Thematic-based reports, on the other hand, were defined as those that described violence against women in broader societal terms by discussing or debating the issue and its antecedents. Results showed that most news reports were episodic (61%, *n* = 271); a minority were thematic (21%, *n* = 93). The most common thematic frames addressed government responses to violence against women or the issue was presented in the context of a political election campaign. A smaller proportion of news reports (18%, *n* = 80) contained elements of both episodic and thematic frames. Table 2 provides a breakdown of reporting frames by media type. News broadcast on radio or television was significantly less likely to report thematically than news reported in print or online (*p* < 0.001).

**Table 2:** Event and thematic reporting of violence against women by media type

	Newspaper		Broadcast		Online		Total
	Frequency (*N* = 93)	%	Frequency (*N* = 221)	%	Frequency (*N* = 130)	%	(*N* = 444)
Event based *only*	36	39	172	78	63	49	271
Thematic *only*	31	33	33	15	29	22	93
Both	26	28	16	7	38	29	80

Across all new reports, a minority included information that explicitly referenced the wider social context in which violence against women occurs (17%, *n* = 76). Among those that did incorporate such contextual features, it was most commonly in the form of prevalence data such as local, state and/or national statistics (91% *n* = 69). Only a small proportion of reports included information about women who may be at higher risk of experiencing violence, such as women with disabilities (21%, *n* = 16) and that violence affects more than individual victims (18%, *n* = 14). The only item referencing the possible impact on children was in the Letters section of one of the capital city newspapers. Less than 5% of reports (*n* = 19) included any information on victim or perpetrator services, such as helplines, websites, advocacy or counseling services. Among the few that did, it was almost always one with a thematic frame (n = 16). Only nine news reports, in total, referred women to a dedicated domestic or family violence telephone helpline service or website. There were no significant differences between media types – with information on help seeking equally unlikely to appear online, in print or within broadcast news.

#### Type of violence

**Fig. 1:**
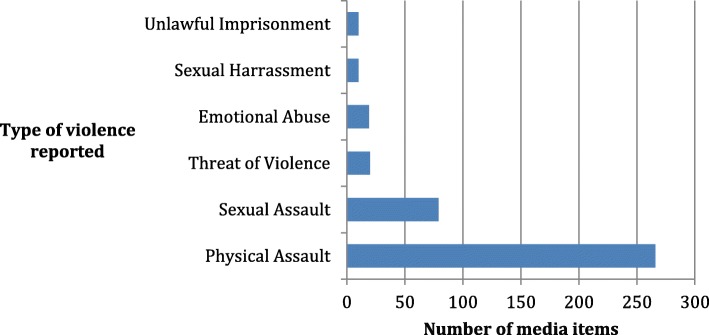
Types of gender-based violence contained in news report (*n* = 351)

Analyses on the type of violence reported in the news was restricted to reports on incidents, leaving a total of 351 news reports. Over three-quarters of reports were in relation to physical violence against women (76%, *n* = 266), followed by sexual violence - sexual assault and/or rape (23%, *n* = 79, see Fig. 1). News reports depicting other types of violent, coercive or controlling behaviours, such as emotional, verbal and financial abuses were rare. We separately analysed the proportion of reports depicting lethal violence and found that 62% were in relation to female homicide (217 of 351 reports).

#### Myths and misconceptions

Using a series of questions, coders identified whether news reports perpetuated common myths and misunderstandings about violence against women, what ‘causes’ violence, and the context in which it occurs. This included, for example, that certain groups in the community (defined by race, religion or class) are more violent than others; that certain circumstances (unemployment, financial strain, addiction) can cause violence or that women are to blame at least partially for violence perpetrated against them. Results showed that only a minority of news reports *explicitly* included misinformation (7%, *n* = 30). This was most commonly in relation to reporting that violence was directly caused by the use or misuse of alcohol and drugs or triggered as a result of an argument or infidelity. No news reports *explicitly* described that violence against women is rare or uncommon, that it is accepted or considered the norm in certain cultures, classes or religions or that perpetrators and victims are equally responsible for violent incidents. However, myths and misconceptions were often implied in news coverage.

Fifteen percent of incident-based reports included information about the behaviour of women (*n* = 51). This included reporting that the female victim was intoxicated (drinking and/or using drugs) at the time of the incident, that she was ‘flirting’ or had gone home with the perpetrator, was out at night or alone, that the victim had provoked the violence, had not reported previous incidents of violence or had stayed with an abusive partner. The same proportion of media reports (15%, *n* = 52) minimised perpetrator blame by portraying that the violence was motivated by love, jealousy, passion or revenge or the use of substances (drugs and alcohol) and thereby uncritically justifying the actions of the perpetrator.

#### Sources of information

Seventy percent (*n* = 311) of all news reports quoted or paraphrased an external source of information. Thematically based reports were more likely to include an external source than reports that were about individual incidents of violence (86% versus 60%, *p* < 0.001). As can be seen in Table 3 the police were the most commonly used external source of information, with close to a third of all news quoting or paraphrasing the police. Overall, legal and criminal justice professionals including police, judges, magistrates and lawyers accounted for half of all sources. Politicians were also a commonly used source of information. Online and broadcast news reports were significantly more likely to quote or paraphrase criminal justice personnel (police, judges, magistrates, lawyers) than newspapers (44, 38, 17%, respectively, *p* < 0.001). Sourcing information from victim/survivor advocates was uncommon across all media types.

**Table 3:** Sources of information quoted or paraphrased (of those that used sources, *n* = 311)^a^

	frequency	%
Police	94	30
Politician	90	29
Perpetrator	34	11
Judge, magistrate or coroner	33	11
Domestic violence advocate and/spokesperson	31	10
Lawyer	28	9
Family of victim	27	9
Victim/survivor advocate	24	8
Other media outlets	18	6
Reports, documents or legislation	14	5
Neighbours or bystanders	13	4
Academics	10	3

^a^Coders selected more than one source (if applicable), therefore frequencies and percentages do not add up to 100

## Discussion

We captured over 4500 unique news reports on violence against women that were broadcast or published over 15,000 times within the study period. The types of stories were broad in scope ranging from short, 30-s radio news clips to lengthy feature articles in large circulation newspapers. The highest volume of reporting was on the radio, which is not surprising given the way news is presented in this medium (i.e. regularly and in high repetition). Of note also is the high volume of reports sourced from online news sites in just two of the four-months in which data was collected. This is significant given the increasingly popularity of and ease of access to digital news. These results confirm that mediated representations of violence against women in the Australian mainstream news are common and thus, offer enormous potential to raise public awareness and disrupt the production and reproduction of gender stereotypes in relation to both cause and consequence.

The study’s findings, together with more recent Australian research [14, 33], suggest that some aspects of mainstream media reporting of violence against women are changing for the better. We note, however, that many of the key areas of concern identified in our work have been raised before, highlighting that progress towards change in journalism practice in this context is slow. While we acknowledge that there isn’t room for in-depth, rich analysis in *every* story, and that legislation can place restrictions on some journalistic freedoms, there remains much opportunity for news organisations to provide their audiences with fair, informative and accurate news on this issue.

In many countries, including Australia, there are self-regulatory mechanisms to guide fairness and accuracy in news writing and reporting on violence against women. Despite that most media guidelines highlight the importance of maintaining a critical connection between individual cases and violence against women as a broader social problem [30], we found that episodic framing continues to be the norm. Episodic framing that depicts violence against women as a series of disconnected, random events is problematic because audiences are more likely to attribute individual blame rather than societal responsibility for the violence [34]. Overall, there were few examples in our data of adherence to media guidelines for reporting on violence against women, with the starkest omission being the lack of information about where to seek help, advice or further support. News reports almost uniformly missed the opportunity to direct their audiences to sources of assistance or community resources. Our study cannot answer the question of why journalists or news organisations do not provide such information. It is unlikely to interfere with the administration of justice in the way that some journalistic practices may, yet it could alert those in need that help is available. The way in which legal processes intersect with journalism in reporting violence against women needs urgent attention and clarification to maximize the potential role of media in prevention of, and responses to, violence against women.

While guides commonly recommend that news reports on violence avoid attributing blame elsewhere (e.g., substance use, financial or other stressors [35]), the issue of drugs and alcohol and its association with the perpetration of violence is complex. While there is no evidence that alcohol is a primary causal agent, there is a strong body of research showing persistent links between the harmful consumption of alcohol and the frequency and severity of violence, particularly domestic and family violence [36]. Media resources and guidelines that simply recommend that media professionals avoid the issue so as not to implicate it as a cause of the violence are not helpful to community understandings about these important and interconnected public health issues. Exploring how news media approaches the issue of violence and alcohol and its impact on audience understandings is an important avenue for future research to pursue.

Additionally, news reports rarely took the opportunity to elevate the voices of survivors, advocates and other experts. Our data confirm previous national and international research showing a disproportionate emphasis by the media on law enforcement and criminal justice perspectives [15, 37, 38]. Although this likely reflects that most news on violence against women is generated from criminal or court proceedings, it has important implications for how the public understands and responds to the issue. International media attention that followed in the wake of the 2016 post on *BuzzFeed* entitled ‘*Here is the powerful letter that the Stanford victim read aloud to her attacker’* [39] highlighted how rarely victim’s voices are heard in media reporting of violence. It was a ‘scoop’ because it was rare for a female victim to be given the means and opportunity to shape the public discourse about her story.

One of the key strengths of the study was that it collected data in connection to all reports on violence against women from a representative sample of mainstream news media in Australia. However, several limitations must be borne in mind when interpreting the findings. First, our sample was restricted to a selection of states, a selection of newspaper outlets in those states and a small selection of online news sites. We note that most sources in our sample were owned by one of two media companies, reflecting the concentration of media ownership in Australia. Copyright issues precluded us from searching some of the more recent overseas entries to the Australian mediascape including *Daily Mail Australia* and the *Guardian Australia*. Second, radio and television reports were only available as a summary broadcast, whereas newspaper and online articles were provided as either full press clippings. This difference may have influenced coding decisions given there was less information available in radio and television broadcasts in comparison to other news reports. While we acknowledge as a limitation that we did not calculate inter-rater reliability estimates at the end of the coding, our processes to ensure consistency were rigorous including that coders were trained in a uniform manner, interpreted data using a detailed coding manual, had regular meetings and discussed difficult and/or contentious coding decisions as a team. Finally, our data represents a contemporaneous ‘snaphot’ of media at a time that coincided with what some other researchers have termed the ‘Rosie Batty effect’ [22]. This refers to a period of time in Australia when Rosie Batty (mother of 11-year-old Luke Batty who was murdered by his father in 2014) rose to prominence as a family violence campaigner influencing public, political and policy debate on the issue. While it is beyond the scope of this study to document her influence on news media reporting, the data are an important source from which to measure change and indeed to test whether the so called ‘Rosie Batty effect’ has been sustained over time.

## Conclusions

Our findings demonstrate that despite the readiness among journalists and readers to engage in news about violence against women, reporting that promotes public understanding of the issue is not always the norm. Many of the key areas of concern highlighted in this study have been raised before and are referred to in Australian and international media and industry guidelines on reporting on violence against women [35]. Their impact, thus far, appears minimal. Reaching all media with a coherent message about reporting practices; however, is notoriously difficult. While there are influential peak bodies in the Australian media industry, they do not enjoy complete regulatory reach. The media are under no direct obligation to adhere to guidelines or recommendations.

Despite these challenges, how the mainstream news media reports violence against women is significant if levels of violence are to be reduced and ultimately eliminated. Media reporting is an important indicator of community attitudes and beliefs about violence against women and thus a critical site through which to measure progress towards shifting social norms that reinforce or exacerbate it [7]. Our findings provide an important avenue to track that progress and to inform current policy initiatives in Australia with a key focus on monitoring progress towards the prevention of violence against women [40].

## Abbreviations

NSW: New South Wales
QLD: Queensland
SA: South Australia
